# Control Aspects of Shape Memory Alloys in Robotics Applications: A Review over the Last Decade

**DOI:** 10.3390/s22134860

**Published:** 2022-06-27

**Authors:** Deivamoney Josephine Selvarani Ruth, Jung-Woo Sohn, Kaliaperumal Dhanalakshmi, Seung-Bok Choi

**Affiliations:** 1 Robert Bosch Centre for Cyber Physical Systems, Indian Institute of Science, Bengaluru 560012, India; rjosephine@iisc.ac.in; 2 Department of Mechanical Design Engineering, Kumoh National Institute of Technology, Daehak-Ro 61, Gumi-si 39177, Korea; jwsohn@kumoh.ac.kr; 3 Department of Instrumentation and Control Engineering, National Institute of Technology Tiruchirappalli, Tiruchirappalli 620015, India; dhanlak@nitt.edu; 4 Department of Mechanical Engineering, The State University of New York, Korea (SUNY Korea), 119 Songdo Moonhwa-Ro, Yeonsu-Gu, Incheon 21985, Korea; 5 Department of Mechanical Engineering, Industrial University of Ho Chi Minh City (IUH), 12 Nguyen Van Bao Street, Go Vap District, Ho Chi Minh City 70000, Vietnam

**Keywords:** shape memory alloy, robotics, actuation, sensing, control

## Abstract

This paper mainly focuses on various types of robots driven or actuated by shape memory alloy (SMA) element in the last decade which has created the potential functionality of SMA in robotics technology, that is classified and discussed. The wide spectrum of increasing use of SMA in the development of robotic systems is due to the increase in the knowledge of handling its functional characteristics such as large actuating force, shape memory effect, and super-elasticity features. These inherent characteristics of SMA can make robotic systems small, flexible, and soft with multi-functions to exhibit different types of moving mechanisms. This article comprehensively investigates three subsections on soft and flexible robots, driving or activating mechanisms, and artificial muscles. Each section provides an insight into literature arranged in chronological order and each piece of literature will be presented with details on its configuration, control, and application.

## 1. Introduction

Shape memory alloy (SMA) is a class of smart material wherein it undergoes changes in its length by contracting to nearly 4% and thereby generates a huge amount of resistance force on its thermal actuation. This property of response to thermal stimuli in this alloy makes it smart, unlike the other alloys. There are types of different compositions of SMAs Ni-Ti alloy (Nitinol), Cu–Al–Ni alloy, Cu–Zn–Al alloy, Au–Cd alloy, Ni–Mn –Ga, and Fe based alloys. Only two alloys that have achieved any level of commercial exploitation are Ni-Ti alloys and copper-based alloys. SMA can operate under two different stimuli, one is thermal stimuli wherein, when a pre-stressed SMA wire (detwinned martensite) undergoes a change in temperature to its safe heating temperature (trained temperature) it remembers its parent shape(austenite) along with the stress it will resume back to its product state and this is called the shape memory effect. This type of feature is used for many position/angle tracking control applications. The other one is stress, where the stimulus at the safe heating temperature acts like a spring enabling it to dissipate a huge amount of energy and making it the right choice for use as dampers and absorbers.

In the early stages of SMA literature, the use of proportional derivatives was handled to operate in the systems but later on, it started to be drift to the implementation of nonlinear controllers or hybrid controllers. The control methods implemented on the SMA-based system are mostly implemented using linear controls if the system demands just the actuation and does not need any precision or accuracy. However, there are complex systems, like when using to an instrument in higher-order systems it demands a nonlinear control for performance and efficacy at higher rates. With a wide understanding of the inherent characteristics, there has been immense growth in terms of literature reports and commercial growth [1,2,3,4,5,6,7]. Position control in shape memory alloy (SMA) has been researched only in the last two decades and it has been growing in different areas starting from the design and development of a servo type of operation using the aSMA element in different configurations and by using different biasing elements. The different aspects of using the element to control position and force for robotic and haptic systems are also treated as principal parameters. Some of the features that are to be noted in developing an SMA-based system are to first to first determine the functionality that is going to operate on the system, composition of the element, structural form, and biasing element. Even though SMA is a non-linear element, there are reports in which it has been noted that the output response of the system remains linear in operation. This behavior is undertaken using an active biasing element which is an antagonistic SMA actuator and the way to make it linear is determined by choosing the inverse mechanical element. In other words, the proper selection of biasing elements can be able to maintain the linearity in the response [8].

The main scope of this article is to timely present a state-of-art on mechanical arrangement of the SMA element along with the biasing, which will eventually provide useful guidelines to design more advanced designs for robotic systems. The control strategies that are mostly and frequently employed in SMA-based robots can be classified into two categories: passive control and active control. In the passive control, ON/OFF control and PWM (pulse width modulation) control are dominant in which the actuating force is converted to the stroke or position of the robotic systems. On the other hand, in the active control, PID (proportional-integral-derivative) control and modified PID control logic such as fuzzy-PID control are frequently used for precise position feedback control of small and soft robots. 

The manuscript has three sections they are flexible/soft robots, drivers and servo actuators, and artificial muscles. At the start of each section there is a brief definition, followed by the literature papers in chronological order stating the mechanism, movement, control, and the application for which it is built.

## 2. Flexible and Soft Robots

Soft robotics were developed using bio-inspired compliance to mimic animal or human capabilities Flexible actuators and electronics are employed to design soft robots. Soft robots are made almost entirely of rigid-body architectures out of flexible, soft material, making them suitable for applications in uncertain, dynamic task environments, including safe human-robot interactions with excellent flexibility and adaptability, but their load capacity is limited [9,10]. The flexibility of SMAs allows us to build actuation components in different configurations and shapes (e.g., helical springs, torsion springs, straight wires, cantilever strips, and torsion tubes), which allow them to be adapted to small, micro, and multi-DOF (degree-of-freedom) applications. Their high force-to-weight ratio and small volume (i.e.) SMA displays one of the highest work densities at 10 J cm^−3^ and can lift more than 100 times its weight—allowing the design of compact and lightweight actuators. 

The generic mechanical design for an SMA-based soft robot is an SMA element, which can be a wire or a spring or any other available configuration followed by a biasing element which in soft robotics will be the chassis material by itself or passive, to enable cyclic operation in the SMA element, and the powering mechanism which is usually a joules heating current. In this section, we can discuss the various design, configurations of SMA elements, and control of the SMA actuator in soft robots [4]. The control is mostly on/off as they focus more on the type of motion to generate using SMA elements.

**Figure 1:**
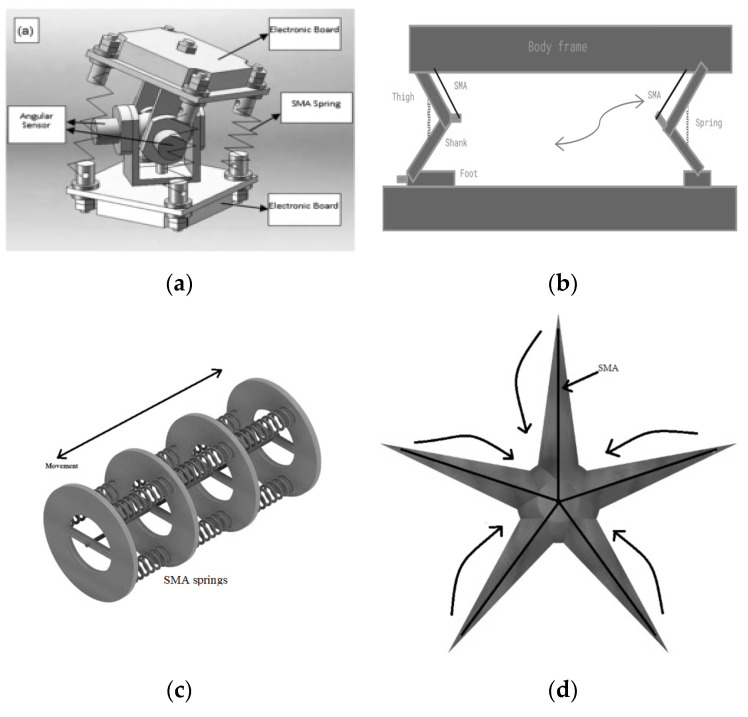

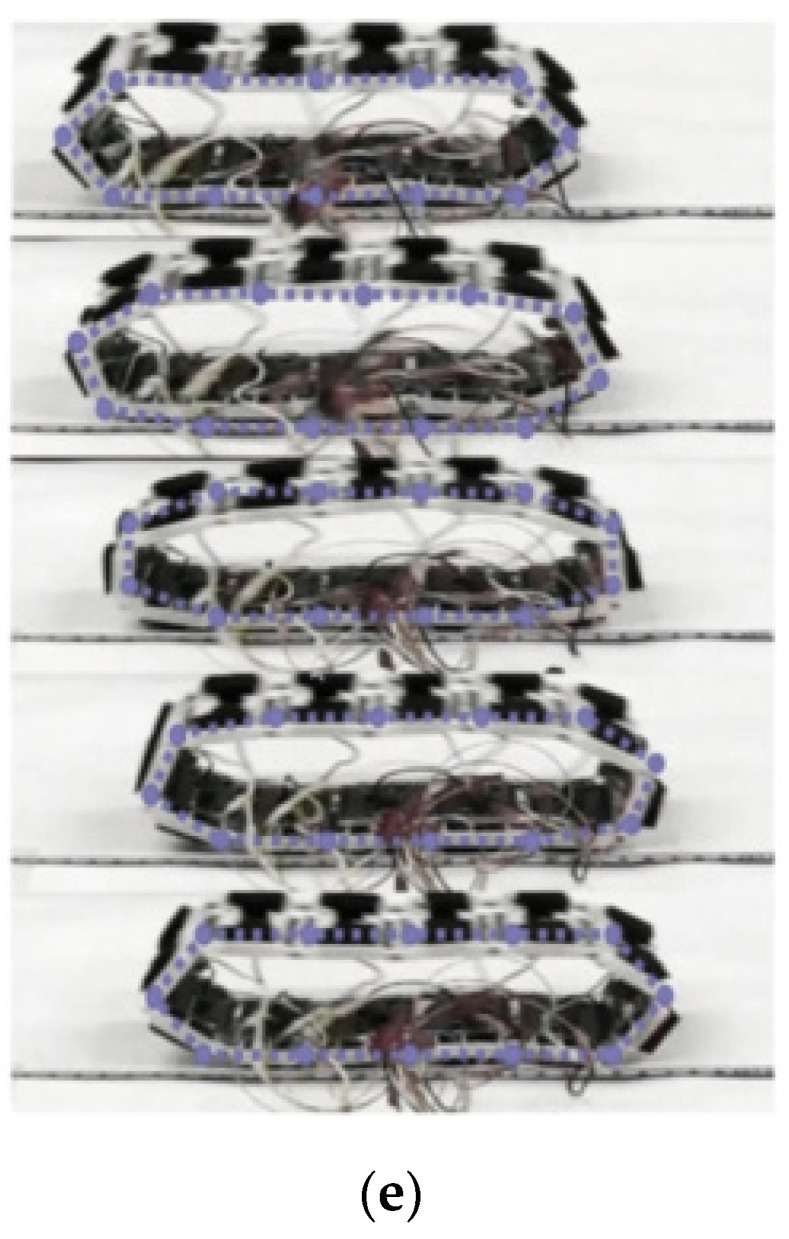
Flexible robots (**a**) fish [10] (**b**) jump-sketch [11] (**c**) crawl -sketch [13] (**d**) star fish-sketch [23] (**e**) roll [41].

In early 2010, FlexiBot (Flexible Robotic Module) was designed with two degrees of freedom and incorporated four memory alloy (SMA) springs as shown in Figure 1a, to create relative motion between two parallel plates hinged to each other providing 30-degree displacements, which make them more suitable for robotic applications [11]. A four-legged robot [12] was created and actuated by SMA wires along with biasing springs to realize jumping motion as in Figure 1b. A finger-sized wood climbing robot [13] with SMA springs can exhibit the crawl, turn, and climb motion on a tree for search and rescue operations. Peristaltic motion [14] was realized by using the parallel configuration of the SMA element for in-pipe movement as in Figure 1c which was designed to crawl for inspection purposes. A snake robot with three links [15] was designed with a PID-fuzzy controller. A biomimetic fish was actuated by SMA wires [16] in enabling bio-inspired locomotion systems using a deformable structure. The fish is controlled with gains set such that the voltage applied to SMA wires has minimum overshoot and the output of the system has minimal time to achieve stability. A novel climbing mode was developed in millirobots built with SMA wire along with a return spring to execute the climbing potential of the robot [17]. A flexible pectoral fin [18] uses two parallel SMA plates, which can perform a bi-directional bending action, and an elastic membrane made of thin rubber, is adhered to the fin rays to function as a bias element. Flea-inspired catapults with SMA springs were used as actuators that can jump more than 200 times their body length with impulse current stimuli [19]. A biomimetic walking microrobot was designed using 11 ICPF (ionic conducting polymer film) actuators to move and two SMA wire actuators to change motion attitude enabling two kinds of motion attitudes: lying state and standing state [20]. A structure with eight SMA springs was developed to have a helical muscular arrangement to simulate the motion of an octopus muscular hydrostat [21]. A compact external pipe crawler robot [22] was designed by deploying a compliant mechanism and SMA actuation that follows clamp-and-push motion and imitates inchworm motion. A soft robot exhibiting sequential antagonistic motion [23] is achieved in a flexible braided mesh-tube structure using nickel-titanium (NiTi) coil actuators wrapped in a spiral pattern around the circumference that exhibits peristaltic locomotion. Starfish-like robots driven by shape SMA spring actuators [24] were designed to accomplish crawling on flat ground, climbing over viscous soil terrain, free motions in random directions, navigating through a target object, and steering as well as grasping imaginary prey as shown in Figure 1d. A flexible microrobot module (FMM) was actuated by SMA springs [25] and able to provide both translational and rotational displacements. Stiquito hexapod mobile [26] robot was designed using antagonist active Nitinol (NiTi) SMA wire/passive music wire couples to produce moving insect-like legs. The starfish-like soft robot with flexible rays using SMA spring [27] with soft silicone material induces multi-gait movements in various environments. BaTboT, a novel bat-like MAV was studied to increase net body forces by implementing with highly articulated wings actuated by shape memory alloy actuators [28]. Soft caudal fin actuators using SMAs [29] that are fixed along with the soft structure of the caudal fin and bend to a certain mode shape can perform steady swimming and maneuvering. The small one DOF mobile robot is actuated by a pair of SMA springs [30], and the developed mechanism can steer in addition to moving forward on a common plane. Bio-inspired multi-arm underwater robotic swimmers actuated by compliant SMA were modeled and developed by actuating spring elements [31]. A locomotive textile-based robotic system was weaved [32] wherein the fabric is integrated with a woven hybrid SMA-textile actuator based designed system. A soft compliant robot [33] exhibiting an inchworm type locomotion was built and tested. Single-caudal fin propelled robot fish using shape memory alloy wire [34] were developed, as well as unique frog-inspired hind limb robots with SMA spring actuators [35] designed to jump. A biomimetic robotic worm was developed to perform a peristaltic motion by employing nine SMA springs in three sections of the soft robot [36]. A flexible parallel robotic module was actuated by three SMA springs in between a triangular top and base plate connected by a universal joint at its centroid [37]. Shape Memory Alloy actuated controllable suction grippers were proposed and experimented with for a wall climbing hexapod [38]. Soft actuators used to perform actions such as bending, twisting and extending using SMA wires were embedded into actuators to power them [39]. A six-legged robot adapting SMA actuators and a spring antagonistic driving mechanism is able to remain at a specific location in the tree without requiring an external energy supply and can walk and climb in a tilted tree at 30 degrees [40]. For the soft robotic arm driven by shape memory alloy (SMA) coils, with a compression compensation algorithm, a proportional-integral differential controller is used to precisely control the two-dimensional motion with a relatively high accuracy [41]. 

SMA-based Roll robot actuators [42] can mimic the behavior of rolling animals as designed in Figure 1e. This is a modular closed-chain rolling robot with compliant SMA wires which has the perfect terrain adaptability and maneuverability. An active Tendril-Backbone Robot (ATBR) was built [43] as the manipulator backbone and actuator which utilized the SMA helix. Fuzzy logic control is implemented to control the displacement by currents for underwater robots in [44]. A scheme to drive multiple flexible fins, was presented and verified the feasibility on a flexible robotic fish driven by SMA wire which is inspired by the swimming mode of devil fish, that was able to achieve more stable motion of the fish, and the movement of the whole fish body was more natural and flexible [45]. A 2DOF soft robotic neck was developed and controlled [46] actuated by a flexible SMA based actuator that allows movements of inclination and orientation. PATRICK, a soft robotic brittle star [47] was the first untethered underwater soft robot using the SMA springs to actuate as in Figure 1e and it was built with a high dimensional actuation space, allowing deeper exploration of planning and control principles. SMALLBug, a crawling microrobot that can locomote at actuation frequencies of up to 20 Hz, was designed, fabricated and tested [48]. The robot is driven by an electrically powered 6 mg bending actuator that is composed of a thin SMA wire and a carbon-fiber piece that acts as a loading leaf-spring and four legs capable of generating anisotropic friction. The papers that reported or designed and developed SMA-based soft robots are presented (in chronological order) in the Table 1 which displays the control handle and the parameters that are measured for the particular application.

## 3. Drivers and Servo Actuations

**Figure 2:**
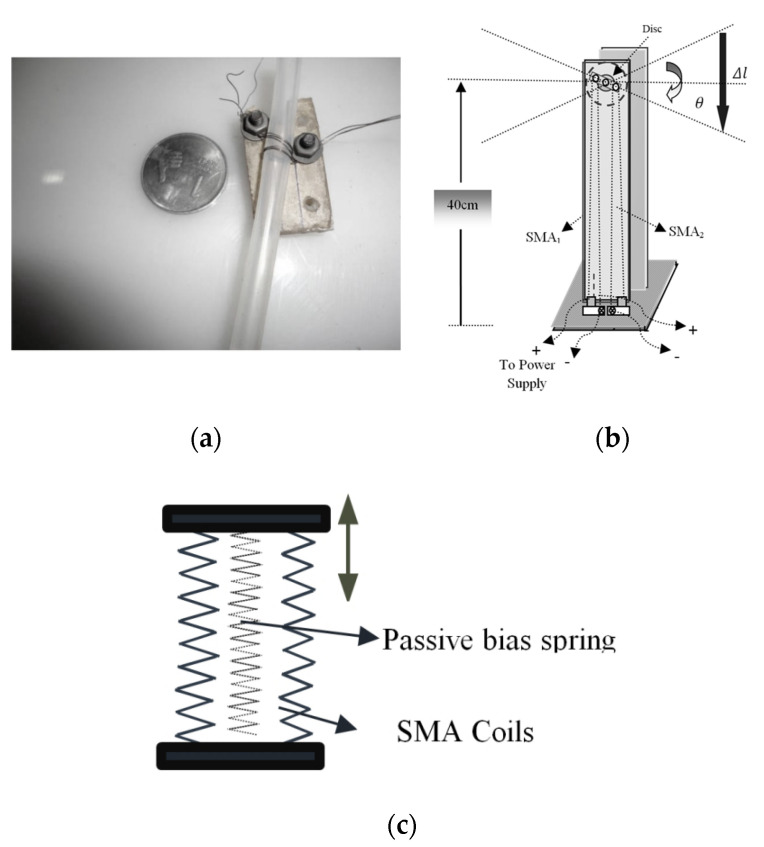
Actuator mechanisms (**a**) flexible pump [52] (**b**) bi-directional servo [54] (**c**) linear actuator–sketch [73].

SMA is an actuator that experiences reduced length enabling a displacement along with force to bring out the work done at that point. Here, the basic element to design is to have an SMA element and a biasing element which would be a passive spring or it can also be active by using SMA elements in an antagonistic configuration to generate a bi-directional movement. The proper design and the understanding of its inherent property changes can enable design of a system with uni-directional or bi-directional linear or rotating movement and any point of application, which proves its use as a driving actuator by substitution in places of traditional classical actuators. An accurate self-sensing method [49] based on the SMA strain to resistance curves for the control of shape memory alloy (SMA) wires biased with passive spring to function as actuated flexures were modeled. An SMA wire actuated gripper was developed [50] to convert the small linear displacement into the angular movement of the gripping fingers to enable open and close functions. A compliant gripper using an SMA coil was fabricated [51] along with a middle flexure joint replicating the behavior of a caterpillar locomotion. A MIniature SwitchAble (MISA) connection system for a stochastic modular robot was designed and implemented [52] which can be switched on and off by controlling four SMA spring actuators. A methodology of actuation to create flow generation in a flexible tube by inducing a variable pressure difference within the tube by external actuation by SMA wires was proposed in [53] shown in Figure 2a. A gripper with soft fingers with 2-DoFs using silicone elastomer rods embedded with shape memory alloy actuators [54], displaying anthropopathic actions was created. 

The sensor-less self-sensing circuit for positioning the 1-DOF manipulator arm using antagonistic self-sensing SMA wires as shown in Figure 2b by implementing fuzzy-PID control was proposed and a real-time experiment was performed [55]. An impact drive mechanism (IDM) using SMA wires for positioning applications was found in [56]. A joint with two degrees of freedom (DOF) driven by antagonistic SMA triple wires using a resistance feedback signal in a closed-loop was designed [57]. SMA wires were characterized to function as a High Phase Order Motor (HPOM) using PWM control [58]. A gripper was designed for a robot arm with an anti-slipping control rule to avoid grabbing an unknown object with insufficient force [59]. A conventional PID controller cascaded with a bilinear compensator, known as BPID, is found to be a promising alternative for controlling the position of the SMA actuator [60]. Antagonistic SMA wires were designed in a configuration to the function as a servomechanism [61] for bidirectional control in a super-articulated system. Self-sensing antagonistic SMA wires were used to establish servo mechanism with bi-directional control in a 1-DOF manipulator arm [62]. A compliant differential SMA actuator [63], composed of two antagonistic SMA wires and a mechanical joint, were coupled with a torsion spring. The master-slave system was set up [64] in which the master is equipped with antagonistic SMA wires to perform the actions to control the 2-DOF slave and also to generate force feedback. A smart soft composite (SSC) hinge actuator using SMA wire in a polydimethylsiloxane (PDMS) matrix was embedded with segmented rigid components capable of a pure bending motion concentrated on specific sections of the actuator [65]. A SMA springs actuated gripper [66] is operated to close and open by applying voltage. A tendon-driven bending actuator [67] using smart soft composite (SSC) and SMA, and a sliding mechanism, which mimics flexion of the human hand were designed. A SMA springs actuated gripper is operated to close and open by applying a voltage as in [68]. Active variable stiffness fibers made from shape memory alloy and thermally responsive polymers that can move to a new position and then hold that position without requiring additional power was designed [69].

A SMA-based soft three-fingered curved gripper [70] was designed which is capable of lifting force nearly three times larger than the gripper. A SMA springs-based soft actuator module (SAM) [71] assembling a connected series of four SAM to develop a soft manipulator was designed, which is capable of three-dimensional spatial grasping motion. Finger-wearable haptic devices [72] for multi-DoF cutaneous force feedback driven by four SMA wires for tip-tilt mechanisms and the planar XY spring with four SMA helixes are employed. An artificial finger [73] is a reproduction of the human finger bone and phalangeal structure, actuated by SMA wires. Shape control [74] of compliant, articulated meshes created from shape memory alloy (SMA)-based linear actuators (Active Cells) capable of ~25% linear strain was explored as shown in Figure 2c. A gecko-like gripper [75] that uses series shape memory alloy (SMA) wire for actuation was created. A compact and modular rotary motor using embedded shape memory alloy (SMA) wire was developed as in [76]. The contraction/expansion of the SMA wires is transmitted as rotational motion that enables the motor to generate continuous rotation and provides higher torque with relatively short-length SMA wires. An antagonistically arranged SMA wire-based actuator was fabricated in [77], which can provide angular displacements in both clockwise and counter-clockwise directions with compliance. Robotic grippers with multiple SMA wires in series along with cross-shear coupler to achieve a larger stroke of actuation were designed [78]. A control method for soft robots on predicting the bending force and RBF compensation to obtain accurate position-tracking performance with adjustable stiffness in both open- and closed-loop control systems was presented in [79].

A continuous bidirectional rotary motor driven by NiTi SMA mini springs was designed in [80]. It is noticeable that its torque/volume and torque/mass ratios are prominent when compared to other motors of the same class. An improved method was based on online data-driven control to drive the robot wrist joint driven by SMA [81]. An Adaptive Neuro-Fuzzy Inference System (ANFIS)-based modeling and control of a 1-DOF modular SMA-based rotary actuator with a compliant motion and fast response was proposed in [82]. A control algorithm for the inversion of the Preisach model for a SMA wire spring-biased actuator under time-varying stress produced accurate results and was computationally efficient was formulated in [83]. A foldable nanosized shape memory actuator into 3D configurations presented in [84] can move around. A numerical was developed for reproducing the mechanical response to integration of the time evolution nonlinear equations governing the response of the SMA spring [85]. The control of a soft planar gripper for grasping deformable objects without integrated sensors, in presented in [86]. The soft finger is a closed-loop PID control system to achieve the desired deformation by introducing a camera as a vision sensor, to detect the bending deformation of the soft finger in real-time. The papers that reported or designed and developed SMA-based actuator-based driving mechanisms are presented in Table 2 which displays the control handle and the parameters that are measured for the particular application.

## 4. Artificial Muscle

SMA actuators, due to their inherent high force to weight ratio feature is an ideal element to replace human muscle, skin, joints, and the skeleton with proper design, configuration, and power units. In this regard, they also found a remarkable place in the development of such a human mimic system. In recent years, our research group has developed a new flexible shape memory alloy actuator that provides more freedom of movement and better integration in wearable robots, especially in soft wearable robots [87]. McKibben developed artificial muscle actuators with shape memory polymers (SMP) [88] to drive robotic joints and these are used in pairs to establish the antagonistic biasing. The wearable supportive device with multiple SMA wires for pulling of the skin (mask) through wires attached to the face as reported in [89].

**Figure 3:**
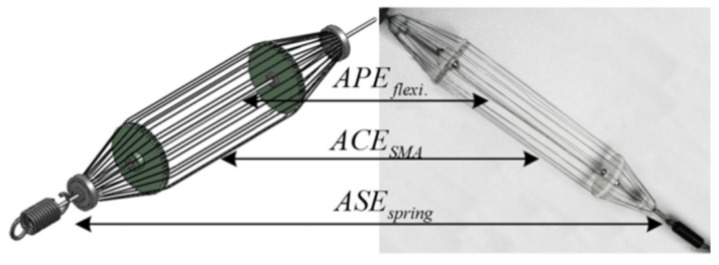
Artificial skeleton muscle [91].

Biomimetic control of a finger actuated by three antagonistic shape memory alloy (SMA) muscle pairs in [90] was designed, where they are each configured in a dual spring-biased configuration by implementing a fuzzy PWM-PID controller. A modified Hysteresis Functional Link Artificial Neural Network (HFLANN) to control an SMA wire actuator [91] was developed. Artificial skeletal muscle (AM) with functions of actuating, energy-storing, and self-sensing using SMA wires and bias spring as shown in Figure 3 was presented in [92]. A single-joint driving system of a bionic finger using pre-shaped SMA wire as the finger skeleton and the joint was designed [93]. To realize bending and stretching of the proposed finger flexibly, a couple of thermoelectric devices (TEDs) were deployed. Impedance control for antagonistic shape memory alloy (SMA) actuators [94] to operate the lower limb exoskeleton was implemented.

Flexible artificial muscle using coiled shape memory alloy (SMA) wires were created [95] to establish bending motion. The possibility of using a parallel arrangement of SMA wires as an actuator in a robotic hand was showcased in [96]. A high-strain flexible actuator using SMA wire that is wrapped around the two pulleys housed inside the Bowden cable sheath for a wrist exoskeleton was designed [97]. A hybrid actuator combining SMA and a DC motor as described in [98] was designed for prosthetic fingers to improve the rate of grasping force rise in the grasping reflex. A robotic hand using SMA springs was developed [99] and by actuating the SMA springs, the fingers can bend or open. The soft robotic hand designed [100] using shape memory alloy (SMA) and woven type smart soft composite (SSC), used 7 DOF in total. Additionally, 11 woven SSC actuators are integrated with soft material as the united structure. A finger-like manipulator [101] operated using antagonistic NiTi SMA wire was reported. Dynamically artificial flower ornaments using SMA wires [102] to perform, the bending of stems, blooming of petals, spreading of fragrance, and flapping of butterflies were developed. The wearable soft grasping support exoskeleton [103], which has a thin and active fixture, is composed of an SMA wire and an air chamber. A biomimetic control method with a 5 × 3 SMA springs array prototype that has characteristics of artificial muscle [104] was framed. The prosthetic finger uses a linkage mechanism creating an underactuated finger motion and driven by an SMA wire actuator to provide high energy density as presented in [105]. The grasping force model for a two-fingered soft robotic gripper [106] using SMA fiber with variable stiffness was developed. It has been noted that quantitatively the kinematics and the static grasping force of the soft finger can be predicted and the grasping force of the soft finger could be adjusted by changing the Young’s modulus of SMA fiber used in the soft finger. The artificial muscle embedded with SMA improves the effective strain of the SMA wires, and thereby improves the artificial muscle modules significantly [107].

Critical issues due to designing a shape memory alloy (SMA) actuation system for a soft robotic finger with a directly 3D-printed stretchable skin-like multilayered tactile sensor [108] were raised. Underwater experiments were conducted using a nonlinear controller to enable precise fingertip force control using feedback from the compliant tactile sensor. A biomimetic 2-DOF SMA-actuated robotic arm [109] controlled by a wearable sleeve in real-time which can mimic users’ shoulders and elbow flexion extension was designed. A muscle-like SMA coil spring, presented in [110], was embedded in the stretchable active coolant circulation system. Modeling of the hand rehabilitation exoskeleton equipment was tested on the index-finger prototype driven by SMA wire, and the finger muscle force was analyzed based on the Hill model as shown in [111]. Bioinspired composite fingers used SMA wires as self-locking joints to perform long-time and high-load grasping tasks with low power consumption as proposed in [112]. An Ionic glove, wearable over a robotic hand, was developed in [113] which contains sensing, computation, and actuation onboard use shape memory alloy (SMA) actuators integrated into an armband to gently squeeze the user’s arm when pressure is sensed in novel electro-fluid. The types of literature that reported or designed and developed SMA-based actuator-based driving mechanisms are presented in Table 3 which displays the control handle and the parameters that are measured for the particular application.

## 5. Conclusions

In this review article, three major subclasses of SMA-based robotic systems were investigated and discussed: soft robots designed with flexible actuators, driving mechanisms to bring out both translational and rotational movement, and vital parts (artificial human parts) for developing some elements to replace the human motor system for rehabilitation or exoskeleton module use. The review analysis of each subclass is summarized as follows. (1) The flexible/soft robots mainly featuring the locomotive-legged kind of robots are most commonly designed and developed. For this, the most commonly used control strategies were traditional on/off control or passive control via the open-loop manner. For the open-loop control, the on/off time remains constant and the speed of operation cannot be changed without programming it. Therefore, the types of robots activated by passive control were functioned to jump, crawl, climb, and roll which can be easily operated by SMA wires/springs in combination with proper biasing elements. (2) In the driving mechanism, the SMA element is employed to independently develop as a mechanism to facilitate movement. Thus, it can either be open or closed and uni-direction as in linear translation and bi-directional movement. In this operation, we need a little more precision when compared to the movement of the soft /flexible robots. One of the widely used controllers in the driving mechanism is the fuzzy-PID controller which can be incorporated with the knowledge of the system. (3) In the development of artificial skin/muscle, the controller must be a closed-loop system so that it can handle real-time movement of human motion and mostly this is designed to be a human interface device. For example, both position and speed should be precisely controlled in the SMA-based wrist exoskeleton mechanism using the feedback controllers such as fuzzy tuned controllers. In this field, to develop more sophisticated human-machine interface devices that should guarantee a higher precision in terms of positioning and generating force, more robust feedback control strategies such as a sliding mode controller need to be implemented for SMA actuators.

It is finally concluded that one of the most significant limitations of application of SMA to various types of robotic systems is a relatively slow response to input stimuli such as current/thermal input compared with other smart material actuators such as piezoelectric ceramic. The response of SMA is closely and directly related to the control bandwidth of application robotic systems exhibiting dynamic movement in a wide frequency spectrum. Recently, to resolve this problem, a new type of SMA activated by magnetic field has been developed, but its application for control of robotic systems is burgeoning.

## Figures and Tables

**Table 1:** Control methods of SMA-based flexible and soft robots.

Control Method	Features/Control Parameter	Application	Reference
Passive control	Heat transfer and constitutive model	FlexiBot	Alireza et al., (2010) [11]
Passive control	Short-time pulse activation	Four-legged robot	Thanhtam et al., (2010) [12]
PWM ^1^	Force to stroke	Omegabot	J. Koh et al., (2010) [13]
PWM	Peristaltic motion mechanism	Micro in-pipe	Gao et al., (2011) [14]
PID ^2^-fuzzy	Position control	Snake robot	Khodayari et al., (2011) [15]
Passive control	Continuous deformable structure	Bio-inspired	Rossi et al., (2011) [16]
Passive contol	Stroke control of a coiled SMA	Millirobots	Kohut et al., (2011) [17]
Passive contol	Curvature—phase transformation	Robotic pectoral fin	Qin Yan et al., (2012) [18]
Passive control	Stiffness to force	Flea inspired catapult	Noh et al., (2012) [19]
Passive control	Motion control	Biomimetic microrobot	Guo et al., (2012) [20]
Passive control	Agonistic-antagonistic	Octopus muscular hydrostat.	Follador et al., (2012) [21]
Passive control	Circumferential motion to ring actuators	Pipe crawler	Singh et al., (2013) [22]
BB and IL ^3^	Iterative learning control	Mesh-worm	Seok et al., (2013) [23]
PP ^4^	Path planning control	Starfish-like robot	Mao et al., (2013) [24]
PID and BB ^5^	Positional asymmetric excitation	Flexible microrobot	Abiri et al., (2013) [25]
Passive control	Periodic current control	Stiquito hexapod	Février et al., (2013) [26]
Sequential control	Kinematic model for motion control to displacement and force	Starfish robot	Shixin et al., (2013) [27]
Passive control	Controlling the modulation of current	Micro-aerial vehicle	Colorado et al., (2014) [28]
PID	Bending curvature control	Caudal fin	Coral et al., (2015) [29]
Passive control	Strain to steer mobility	Mobile robot	Hadi et al., (2015) [30]
Closed loop controller	Speed and force	Robotic swimmer	Sfakiotakis et al., (2015) [31]
Passive control	Force coupled with displacement	Textile robots	Kennedy and Fontecchio (2017) [32]
PWM	Differential friction	Inchworm robot	Pillai et al., (2017) [33]
Passive control	Acceleration and angular velocity	Robotic fish	Li and Li (2017) [34]
Passive control	Passive force to length of wires	Frog like robot	Ren et al., (2017) [35]
PWM Control	peristaltic motion and the orientation	Soft robot	Alcaide et al., (2017) [36]
ON/OFF control	Liang dynamic model	Flexible SMA actuators	Ranjith et al., (2018) [37]
Open-loop position control	Shear stress control	Legged and non-legged	Avadhoot et al., (2018) [38]
Open-loop testing	Finite element model	Soft gripper	Saeed et al., (2019) [39]
Passive control	Deformation and torque for roll yaw directions	Legged robots	Ishibashi et al., (2019)[40]
PID controller and CCA ^6^	Bending movement	Soft robots	Yang et al., (2019) [41]
Passive control	Improved mobility and good terrain adaptability	Rolling robots	Nader et al., (2020) [42]
Passive control	Bending angles—angular speed	Continuous manipulator	Sonaike et al., (2020) [43]
Simulation	3D motion	Bionic Devil Fish	Chen and Liu (2020) [44]
BPID ^7^	Inclination and orientation	Soft robotic neck	Copaci et al., (2020) [45]
MP and GPA ^8^	Applied current to bending	Underwater robots	Cruz et al., (2020) [46] Patterson et al., (2020) [47]
Passive control	High-speed thermally-induced transformations	SMALLbug	Nguyen et al., (2020) [48]

^1^ Pulse Width Modulation, ^2^ Proportional-Integral Derivative, ^3^ Bang Bang and Interative Learning, ^4^ Path Planning, ^5^ Bang bang, ^6^ Compressing Compensating Algorithm, ^7^ Bilinear Proportional Integral Derivative, ^8^ Motion Planning and Greedy Planning Algorithm.

**Table 2:** Control methods of SMA as driving mechanisms.

Control Method	Features/Control Parameter	Application	Reference
Passive control	Strain to resistance modeling	Gripping fingers	Chao-Chieh et al., (2010) [49]
Passive control	Linear into angular movement	Three-fingered gripper	Khodayari et al., (2011) [50]
Passive Contol	Gripping force changes with the length of the flexure joint	Bio-inspired gripper	Gwang-Pil et al., (2011) [51]
Passive control	Differential actuation system	Connection	Guoqiang et al., (2012) [52]
Passive control	Variable pressure difference	Displacement pumps	Keerthi et al., (2013) [53]
Passive control	Gripping force distribution between the finger and the object	Soft robot gripper	Obaji and Zhang (2013) [54]
Fuzzy-PID control	Strain to differential resistance	1-DOF manipulator arm	Josephine et al., (2013) [55]
PI control	Bidirectional strain/displacement to step movement	Positioning device	Shinya et al., (2013) [56]
Fuzzy-PID control	Resistance feedback	Ball joint for end effector	Zhenyun et al., (2014) [57]
PWM control	Enhancement of force and control	SMA based motor	Rossi et al., (2014) [58]
Fuzzy sliding-mode control	Anti-slip control by force sensing	Robotic gripper	Shaw and Lee (2014) [59]
PID controller cascaded with a BPID	Position control	Position control	Álvaro et al., (2015) [60]
Fuzzy-SMC	Strain to position control	Ball balancing beam	Sunjai et al., (2015) [61]
		(underactuated)	
Sliding mode control	Strain to differential resistance	1-DOF bidirectional servo actuation	Josephine et al., (2015) [62]
PI and saturated PI	Stiffness and compliance	Servomechanism	Zhao et al., (2015) [63]
PD control	Electrical resistance and force feedback (haptics)	Master-slave systems	Josephine et al., (2016) [64]
Passive control	Pulling and grasping	Three-fingered gripper	Wei et al., (2016) [65]
Passive control	Bending and load holding	Robotic hand	Hyung et al., (2016) [66]
PWM	Close and open	Gripper	Rad et al., (2016) [67]
Passive control	Actuation and variable stiffness	Robotic skin	Yuen et al., (2016) [68]
Passive control	Thermoconstitutive model deformation of the actuator	Curved gripper	Hugo et al., (2017) [69]
Higher-order SMC	Differential electrical resistance	1-DOF manipulator arm	Josephine et al., (2017) [70]
PWM	SMA resistance, self-feedback	Soft manipulator	Zhang et al., (2017) [71]
Passive control	Touch/pressure—shearing force	Haptic device	Lim et al., (2017) [72]
Passive control	Extension and flexion force	Prosthetic hand	Van der et al., (2017)[73]
PD control	Shape control based linear actuators -Active Cells	MACRO	Nawroj et al., (2017) [74]
Passive control	Adhesive pressure control	Gecko inspired gripper	Mehdi et al., (2018) [75]
Open-loop testing	Continuous and bidirectional rotation	Wearable rehabilitation	Hwang et al., (2018) [76]
PID control	Angular displacements with compliance	Soft bio-inspired robotic systems	Youngshik et al., (2019) [77]
Open-loop testing	Theoretical model of grasping force for different capturing targets.	Robotic gripper	Yifan et al., (2019) [78]
Radial basis function (RBF) + SMC	Two different position controls	Soft robot	Junfeng (2019) [79]
Open-loop control	Numerical and experimental responses of angular displacement, force, and torque	Servo drive (motor)	José et al., (2020) [80]
Data driven control	Displacement control	Rehabilitation medical devices	Zhang et al., (2020) [81]
ANFIS	Closed-chain serial mechanism	Bio-inspired and soft robotics	Mansour et al., (2020) [82]
Open-loop control	Active cooling system for efficient response	Wearable robotics	Joey et al., (2020) [83]
Open-loop control	Curvation variation	Foldable robot	Cordelia (2021) [84]
Backward Euler time integration algorithm and the prediction-correction technique	Euler time integration algorithm and the prediction-correction technique	SMA actuator	Esposito et al., (2021) [85]
PID control	Gripping force	Soft gripper	Wei et al., (2021) [86]

**Table 3:** Control methods of SMA as artificial muscle and finger.

Control Method	Features/Control Parameter	Application	Reference
Passive control	Tension to length relationship	Robotic arm joints	Kazuto et al., (2010) [88]
PID controller	Displacement/Strain	Robot mask system	Jayatilake et al., (2010) [89]
Fuzzy PWM-PID	Bi-directional motion	Anthropomorphic artificial finger	Junghyuk et al., (2011) [90]
Predictive control	HFLANN	Linkages	Nguyen et al., (2012) [91]
Fuzzy tuned PID controller	Force–velocity and force–length relationships	1 DOF robotic ankle-foot	Jianjun et al., (2012) [92]
PI controller	Strain to bending angle	Bionic finger	Sun et al., (2012) [93]
PID controller	Impedence control	Exoskeletons	Araujo et al., (2012) [94]
Passive control	Bending angle	Flexible Artificial Muscle Actuator	Hironari (2013) [95]
adaptive PID	Hysteresis-prone phase transition	Robotic hand	Gerrit et al., (2015) [96]
Hammerstein-Wiener modeled PID gains	Position and speed control	Wrist exoskeleton	Villoslada et al., (2015) [97]
Passive control	Improving reflex speed by controlling voltage	Prosthetic finger	Fei Gao et al., (2015) [98]
Passive control	Strain to bending angle	Prosthetic finger	Ahmadi et al., (2015) [99]
Passive control	Bending curvature control	Bio-mimetic soft hand.	Kim et al., (2015) [100]
Passive control	Thermal setting technique	Robotic finger	Dilibal et al., (2015) [101]
PWM	Deflection control	Artificial flowers	Pan et al., (2015) [102]
Passive control	Holding/grasping force	Grasping support exoskeleton	Hasegawa and T. Suzuki (2015) [103]
Programmable logic controller	Displacement and Force	Artificial muscle	Ying et al., (2015) [104]
Passive control	Underactuated finger motion	Robotic finger	Lee et al., (2016) [105]
Characterization	Cosserat theory-based grasping force model	Soft robotic gripper	Yin et al., (2018) [106]
Open-loop tension tests	Strain and weaving angle correlation	Artificial muscle modules	Kong et al., (2018) [107]
PID control	Precise fingertip force control using feedback from the compliant tactile sensor	Underwater gripper	Maohua et al., (2020) [108]
PID controller	Joint angular position	Rehabilitation, haptics, and, surgical robotics	Golgouneh et al., (2020) [109]
Open-loop control	Active cooling system for efficient response	Wearable robotics	Jeong et al., (2020) [110]
PWM	Coupling dynamic model for modeling and analyze	Exoskeleton	Wang et al., (2020) [111]
Open-loop control	Self-locking joints	Assisting UAV for perching and grasping bio-inspired finger	Hu et al., (2021) [112]
Open-loop control	Intuitive grasping	Prosthetic hand	Simons et al., (2021) [113]

## Data Availability

The raw/processed data required to reproduce these findings cannot be shared at this time as the data also form part of an ongoing study. In the future, however, the raw data required to reproduce these findings will be available from the corresponding authors.
